# ThicknessTool: automated ImageJ retinal layer thickness and profile in digital images

**DOI:** 10.1038/s41598-020-75501-y

**Published:** 2020-10-28

**Authors:** Daniel E. Maidana, Shoji Notomi, Takashi Ueta, Tianna Zhou, Danica Joseph, Cassandra Kosmidou, Josep Maria Caminal-Mitjana, Joan W. Miller, Demetrios G. Vavvas

**Affiliations:** 1 From the Retina Service, Angiogenesis Lab, Massachusetts Eye and Ear Infirmary, Harvard Medical School, Boston, MA USA; 2 From the Department of Ophthalmology and Visual Sciences, University of Illinois at Chicago, Chicago, IL USA; 3 From the Retina Service, Bellvitge Hospital, University of Barcelona, Barcelona, Spain

**Keywords:** Cell death, Computational science

## Abstract

To develop an automated retina layer thickness measurement tool for the ImageJ platform, to quantitate nuclear layers following the retina contour. We developed the ThicknessTool (TT), an automated thickness measurement plugin for the ImageJ platform. To calibrate TT, we created a calibration dataset of mock binary skeletonized mask images with increasing thickness masks and different rotations. Following, we created a training dataset and performed an agreement analysis of thickness measurements between TT and two masked manual observers. Finally, we tested the performance of TT measurements in a validation dataset of retinal detachment images. In the calibration dataset, there were no differences in layer thickness between measured and known thickness masks, with an overall coefficient of variation of 0.00%. Training dataset measurements of immunofluorescence retina nuclear layers disclosed no significant differences between TT and any observer’s average outer nuclear layer (ONL) (*p* = 0.998), inner nuclear layer (INL) (*p* = 0.807), and ONL/INL ratio (*p* = 0.944) measurements. Agreement analysis showed that bias between TT vs. observers’ mean was lower than between any observers’ mean against each other in the ONL (0.77 ± 0.34 µm vs 3.25 ± 0.33 µm) and INL (1.59 ± 0.28 µm vs 2.82 ± 0.36 µm). Validation dataset showed that TT can detect significant and true ONL thinning (*p* = 0.006), more sensitive than manual measurement capabilities (*p* = 0.069). ThicknessTool can measure retina nuclear layers thickness in a fast, accurate, and precise manner with multi-platform capabilities. In addition, the TT can be customized to user preferences and is freely available to download.

## Introduction

Progressive photoreceptor cell death is a significant culprit in retinal degenerative diseases. Inasmuch, quantitation of this cell loss has been addressed by a myriad of approaches, predominantly in experimental models of retinal diseases, such as retinal detachment^1,2^. Among these methods, photoreceptor cell death assays^3,4^, outer nuclear layer cell counting^5^, and outer nuclear layer thickness^6,7^, have been used in animal models to quantitate photoreceptor degeneration. Given the technical and time constraints related to cell death assays and manual counting; to date, outer nuclear layer (ONL) thickness has been widely used as a practical proxy method to estimate the depth of photoreceptor cell death^6–8^.

The utility of retina ONL thickness quantitation to infer photoreceptor degeneration relies on manual calliper measurements. However, the advantage of this approach can be compromised as callipers are manually drawn and measured by an observer. In addition, given the curvature of the retina, manually positioning callipers perpendicular to the layer contour along the long axis can be difficult. Furthermore, given the high magnification of microscope images, a small area of interest is often analysed to expedite the analysis, which can lead to bias. As new imaging modalities also become available, a versatile method that can adapt to multiple imaging modalities is ideal. Therefore, a tool that can adapt to layer architecture and contour to measure thickness in a broad segment, in multiple layers, in either single images or large tiles, and in multiple platforms, is very compelling. Despite pioneering work done in this area^9^, to the best of our knowledge, there is currently no freely available tool for the ImageJ platform to automatedly quantitate multiple layer thicknesses in large images and in different imaging modalities.

The purpose of this work was to develop an automated retinal layer thickness measurement tool for the ImageJ platform, which can quantitate nuclear layers following the retina contour, with callipers as close to 1-pixel to each other. For this purpose, we developed the ThicknessTool (TT) and validated its accuracy by objective calibration and agreement analysis with two masked observers. We found that this measurement tool can provide accurate and precise thickness measurements. In addition, TT can process images from multiple imaging modalities.

## Materials and methods

All animals used in experiments and breeding adhered to the statement of the Association for Research in Vision and Ophthalmology (ARVO). Animal protocols were reviewed and approved by the Animal Care Committee of the Massachusetts Eye and Ear Infirmary. To validate the ThicknessTool in experimental model, a retinal detachment was induced in eight-week-old C57BL/6J mice, as previously described^10^. C57BL/6J mice were purchased from The Jackson Laboratories and maintained in a standard 12-h light/dark cycle.

### Digital image dataset

#### Calibration dataset

We created an image dataset of fifty 8-bit mock images of 1344 × 1024 pixels containing masks of increasing thickness, from 10 to 500-pixel thickness with 10-pixel increments. Following, we created a second dataset of images with a known mask area of 1344 × 200 pixels with 10° rotation intervals, plus 45° and 135° rotations for a total of 21 images. Finally, to emulate layer thinning and thickening, we created a single mock image containing a mask with abrupt thickness changes, with segments of 200, 150, and 250 pixels. All images were stored in TIFF format, and the measured thickness was compared to the known standard.

#### Calibration criteria

We defined the overall mean thickness limit of agreement to 1 pixel, and the following criteria for the ThicknessTool script calibration: (1) overall mean thickness equal to 200 ± 1 pixel; (2) index of dispersion equal to 0, as the given area is a rectangle and all callipers should be parallel to each other with equal thickness; (3) single calliper measurement at 0°, 45°, 90°, 135°, and 180° rotations equal to 200 pixels; and (4) single calliper measurement equal to 200 pixels ± 2 pixels at the remaining rotations. This difference corresponds to the aliasing effect on area edges at rotations other than the specified on (3). The occurrence of uneven jagged edges requires a broader margin of error (2 pixels) than images with straight (0°, 90°, 180°) or even (45° and 135°) edges. For comparisons, the measured mean was tested against a hypothesized mean of 200 pixels. An equivalence test with a two one-sided tests (TOST) approach was used to assess minimum and maximum single calliper measurements, and the threshold difference considered equivalent to no difference was defined as 1 for images with even edges and 2 for images with uneven edges.

#### Training dataset

Immunofluorescence digital images of 4′,6-diamidino-2-phenylindole (DAPI)-stained retinal cross-sections were obtained from the Angiogenesis Laboratory fluorescence microscopy database. We selected images from a murine retinal detachment model in order to evaluate TT performance with different ONL thicknesses and various retinal distortions. A total of 64 randomly selected images were included in this dataset and stored in TIFF format for further processing. Training dataset images were measured by an inexperienced observer who never performed the given task before, and by an experienced observer who had performed these measurements before. Images of murine retina displaying the outer (ONL) and inner nuclear layer (INL) were masked and given to observers. Each image was measured twice in a masked manner. Observers measured each layer with six individual callipers, placed across the retinal layer at their discretion. Single callipers were used to calculate mean, minimum, and maximum layer thickness.

#### Validation dataset

We randomly selected 16 images from 8 eyes of an experimental murine retinal detachment model. The layer thickness was manually assessed by an observer, who measured ONL and INL in the detached and attached retina, with six individual callipers for each layer.

### Thickness script

We developed a script for the ImageJ platform (version 1.52p, https://imagej.nih.gov/ij/; provided in the public domain by the National Institutes of Health, Bethesda, MD, USA). To facilitate the use of the script, we designed a graphical user interface dialog to tailor performance parameters to users’ preferences, including image scale, number of callipers, skeleton width, among others (Supplementary Fig. 1).

**Figure 1:**
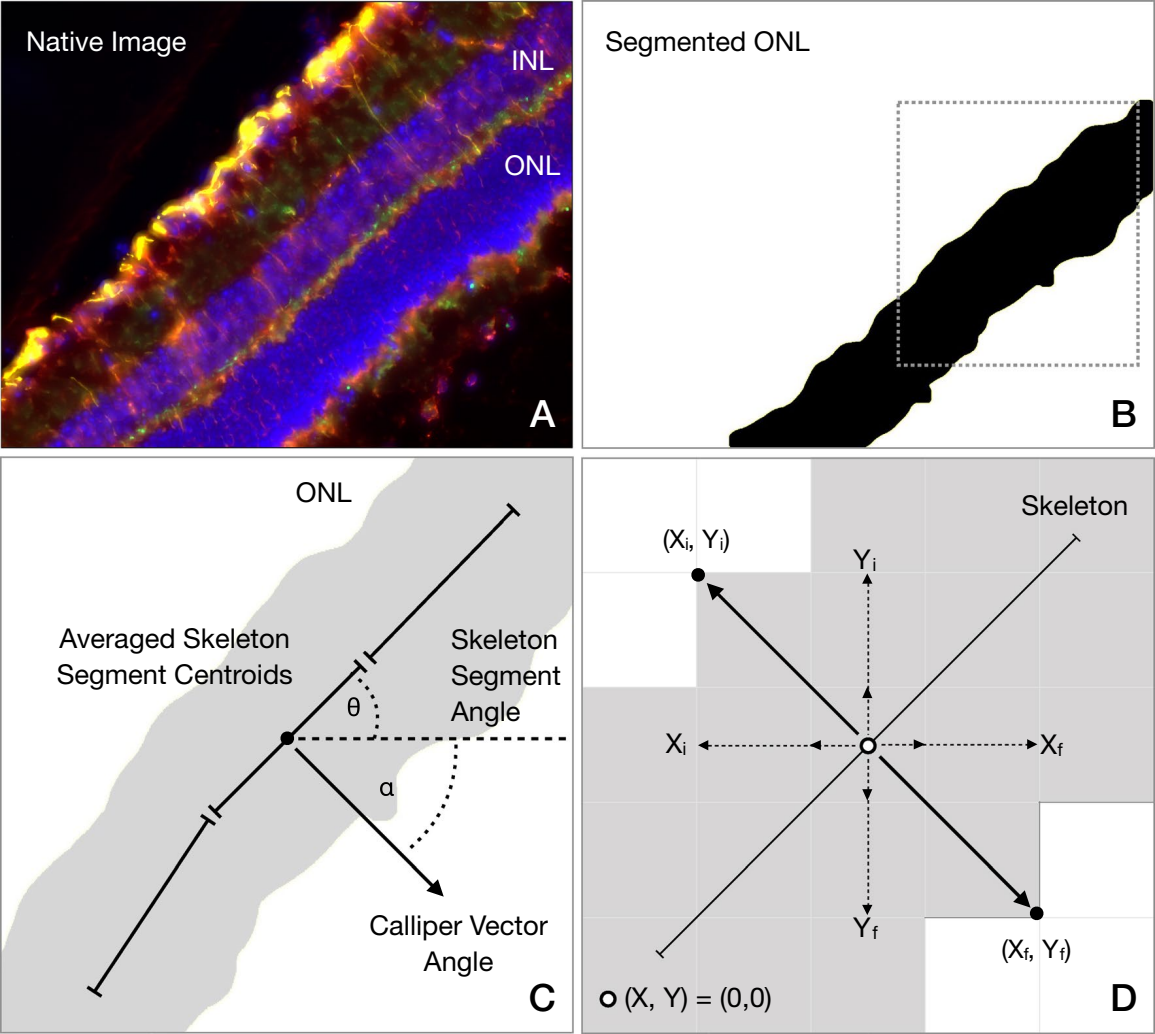
Outline of automated segmentation, thickness calliper constructs, and thickness measurements by ThicknessTool. (**A**) Representative native image acquired from mouse retina section and immunostaining. (**B**) Segmented outer nuclear layer (ONL) and mask. (**C**) Cropped area of the outer nuclear layer (ONL) mask. The angle of each skeleton segment (θ) was calculated respective to the zero-plane. The calliper angle (α), and resulting vector, were calculated as the supplementary angle of (θ). (**D**) Digital image representation displaying a pixel-grid and averaged skeleton segment calculated for the ONL mask, resulting vectors, and calliper endpoints.

Briefly, retinal layers were segmented using a previously validated image processing algorithm (Fig. 1A–D)^4^. From segmented layers, a skeletonized mask was created with AnalyzeSkeleton function, as previously described by Arganda-Carreras and coauthors^11^. Following, thickness callipers were constructed as follows (Fig. 1D): (1) The segmented mask skeleton was fragmented in segments by user-defined *n* number of callipers; (2) Centroids from these segmented skeletons were isolated as single points and defined by the coordinates (X_0_, Y_0_); (3) The skeleton segment angle was calculated by an user-defined grid which evaluated the angle of the segmented mask skeleton in a given area; (4) From each centroid (X_0_, Y_0_), a positive final vector and a negative initial vector were created perpendicularly to the segmented skeleton. The initial and final vectors were composed by a corresponding horizontal component vector (X_*step*_), and a vertical component vector (Y_*step*_); (5) The X_*step*_ and Y_*step*_ components were created for the initial and final resolved vector at a 1-pixel distance from the centroid in horizontal and vertical directions, respectively. The preliminary initial (X_*i*_, Y_*i*_) and final (X_*f*_, Y_*f*_) coordinates were created from the centroid as described in Eq. (1); (6) Pixel values were evaluated in the retinal layer mask at *initial* (X_*i*_, Y_*i*_) and *final* (X_*f*_, Y_*f*_) coordinates. If the pixel value returned *zero*, this was interpreted as the retinal layer mask, and the X_*step*_ and Y_*step*_ components were increased by 1-pixel, to analyse the contiguous pixel; (7) This process was iterated until the pixel value was > 0, and this point was assumed as the *initial* (X_i_, Y_i_) and *final* (X_f_, Y_f_) vector endpoint; (8) Thickness was calculated as the rounded Euclidean distance between *initial* (X_i_, Y_i_) and *final* (X_f_, Y_f_), as seen in Eq. (2).

1 $$\begin{gathered} X_{i} = round\left( {\sin \left( {\alpha \times \frac{\pi }{180}} \right) \times X_{step} + X_{0} } \right) \hfill \\ Y_{i} = round\left( {Y_{0} - \cos \left( {\alpha \times \frac{\pi }{180}} \right) \times Y_{step} } \right) \hfill \\ X_{f} = round\left( {X_{0} - \sin \left( {\alpha \times \frac{\pi }{180}} \right) \times (X_{step} - \sqrt {1 + Y_{step}^{2} } )} \right) \hfill \\ Y_{f} = round\left( {\cos \left( {\alpha \times \frac{\pi }{180}} \right) \times (Y_{step} - \sqrt {1 + Y_{step}^{2} ) + } Y_{0} } \right) \hfill \\ \end{gathered}$$

2 $$Thickness Caliper = \sqrt {(X_{f} - X_{i} )^{2} + (Y_{f} - Y_{i} )^{2} }$$

where *i* = initial; *f* = final; α = skeleton supplementary angle; *X*_*0*_ = x-axis centroid coordinate; *Y*_*0*_ = y-axis centroid coordinate; *X*_*i*_ = *initial* horizontal vector component endpoint x-axis coordinate; *Y*_*i*_ = *initial* vertical vector component endpoint y-axis coordinate; *X*_*f*_ = *final* horizontal vector component endpoint x-axis coordinate; *Y*_*f*_ = *final* vertical vector component endpoint y-axis coordinate; *X*_*step*_ = 1-pixel increment in the x-axis; *Y*_*step*_ = 1-pixel increment in the y-axis.

### Statistical analyses

Statistical analysis was performed with SAS software (2019 SAS, NC). Normality was assessed with Shapiro–Wilk test. Statistical significance for differences between groups was determined with t-test for matched pairs or Wilcoxon signed-rank test for matched pairs, t-test for independent samples, and one-way ANOVA with Tukey post hoc correction for multiple comparisons. For correlation analysis, values were expressed as Pearson correlation coefficient (*r*) and statistical significance. Parallelism plots were constructed fitting a cubic spline with a default lambda of 0.05. Agreement analysis was performed by Bland–Altman plot method^12,13^, and as previously described^4^. Coefficient of variation (CoV) was calculated as the difference between measurements over their mean [(A – B)/(A + B)/2] and CoV% as CoV*100. The index of dispersion was calculated as variance-to-mean ratio. Results are expressed as mean ± standard deviation (SD). A *p* value of < 0.050 was considered statistically significant.

## Results

### ThicknessTool calibration

To calibrate the ThicknessTool algorithm, we created three mock image datasets with masks of known area thickness and rotation. First, the TT was able to accurately measure areas of increasing thickness, from 10 to 500 pixels, with a bias of 0.00 ± 0.00 (Supplementary Fig. 2) and thus, as expected, the correlation coefficient for the known vs. measured thickness was 1.00 (*p* < 0.001). Following, we evaluated the performance of the algorithm at different layer rotations with 200-pixel masks at different angles. We predefined the known or theoretical mean, minimum, and maximum thickness to 200 pixels, a coefficient of variation (CoV) of 0.00, and a margin of error of 1-pixel. As seen in Supplementary Table 1, the mean thickness measured by ThicknessTool was 199.88 ± 0.25 pixels, with a CoV of 0.00 for the overall sample. There were no statistically significant differences between the measured overall mean thickness and hypothesized mean (*p* = 0.06). Mean thickness for images with even and jagged edges fulfilled the calibration criteria as previously defined, with CoV of 0.00 (Supplementary Fig. 2A–D). Equivalence Test of mean, minimum and maximum calliper thickness rejected both null hypotheses (both *p* < 0.001), indicating that the observed difference does not exceed the defined threshold. Finally, we tested TT capability to detect true thinning and thickening in a mock image with decreasing and increasing thickness, respectively. TT accurately measured varying thickness (*r* = 1.00, *p* < 0.001), with a bias of 0.00 ± 0.00 and no significant difference between known vs. measured markers (*p* = 1.000) (Supplementary Fig. 2F). These results indicate that the ThicknessTool can automatedly measure the different thicknesses and at different rotations in an accurate and precise manner.

### Training dataset mean layer thickness

**Figure 2:**
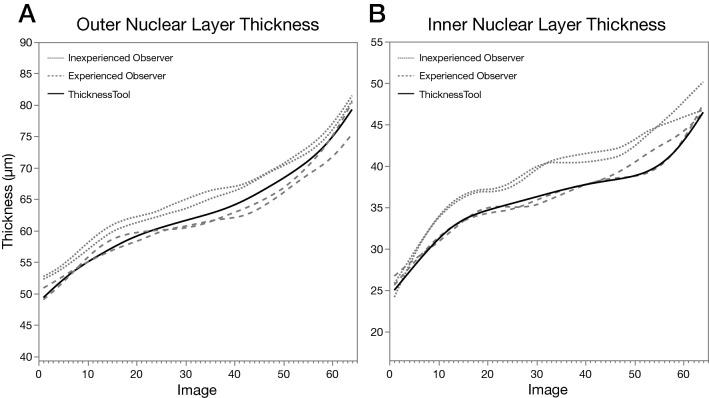
Parallelism plot of mean retinal thickness measurements of observers and ThicknessTool. Results were fitted to a cubic spline with a default lambda of 0.05. (**A**) Outer nuclear layer. (**B**) Inner nuclear layer. Dotted lines represent the first and second measurements by an inexperienced observer. Dashed lines represent the first and second measurements by an experienced observer. Solid lines represent measurements by ThicknessTool. Images are sorted by ThicknessTool values in ascending order (n = 64).

To evaluate the performance of observers and TT across different images and thickness, we first constructed a parallelism plot of mean ONL and INL thickness. As seen in Fig. 2, the inexperienced observer’s mean measurements were consistently higher than the experienced and TT, which in return were similar and parallel to each other in both ONL and INL layers, suggesting a proportionate performance. Correlation coefficient (*r*) analysis between TT and observers’ measurements were > 0.88 (*p* < 0.001) for the ONL and > 0.84 (*p* < 0.001) for the INL (Supplementary Table 2 and 3).

The mean ONL thickness was 65.26 ± 7.78 µm, 62.00 ± 7.14 µm, and 62.85 ± 7.33 µm for the inexperienced, experienced, and TT, respectively (Table 1). There were no significant differences between TT and any observer or average ONL measurements. The mean INL thickness was 39.13 ± 6.22 µm, 36.31 ± 5.17 µm, 36.12 ± 4.72 µm for the inexperienced, experienced and TT, respectively. In this group, there was only a significant difference between the inexperienced first measurement and TT (39.23 ± 6.30 µm vs. 36.12 ± 4.72 µm, *p* = 0.044), and no significant differences between TT and any observer average INL measurements. The average for the inexperienced vs. experienced was not significantly different, in either the ONL (*p* = 0.219) and INL (p = 0.097), with overlapping confidence intervals. Moreover, there were no significant differences between TT and any observer or average ONL/INL ratio measurements, with overlapping 95% confidence intervals. Altogether, these results suggest that no significant difference was evidenced regarding the observers’ prior experience. However, TT ONL and INL measurements stand more closely to experienced observer’s results. Moreover, TT measurements are not significantly different from any observers’ ONL and INL average.

**Table 1:** Training dataset mean thickness measured by observers and ThicknessTool.

	Outer nuclear layer			Inner nuclear layer			ONL/INL ratio	
	Mean ± SD	95% CI	*P* value*	Mean ± SD	95% CI	*P* value*	Mean ± SD	*P* value*
Inexperienced 1st	65.69 ± 7.94	63.70–67.67	0.394	39.23 ± 6.30	37.66–40.80	**0.044**	1.70 ± 0.26	0.910
Inexperienced 2nd	64.83 ± 7.78	62.88–66.77	0.814	39.03 ± 6.33	37.45–40.61	0.078	1.69 ± 0.26	0.768
Inexperienced Average	65.26 ± 7.78	63.31–67.20	0.613	39.13 ± 6.22	37.57–40.68	0.059	1.70 ± 0.26	0.832
Experienced 1st	62.44 ± 7.72	60.51–64.37	1.000	36.50 ± 5.70	35.07–37.92	1.000	1.74 ± 0.26	0.999
Experienced 2nd	61.56 ± 6.83	59.84–63.26	0.978	36.12 ± 5.29	34.79–37.44	1.000	1.73 ± 0.21	0.994
Experienced Average	62.00 ± 7.14	60.21–63.78	0.998	36.31 ± 5.17	35.01–37.60	1.000	1.73 ± 0.21	0.996
Observers average	63.47 ± 7.60	61.79–65.46	0.998	37.64 ± 5.68	36.34–39.10	0.807	1.71 ± 0.22	0.944
ThicknessTool	62.85 ± 7.33	61.01–64.68	–	36.12 ± 4.72	34.94–37.30	–	1.76 ± 0.22	–

Values shown are means ± standard deviations.

*ONL* outer nuclear layer, *INL* inner nuclear layer, *SD* standard deviation, *CI* confidence interval, *1st* first measurement, *2nd* second measurement.

*Comparisons for all pairs vs ThicknessTool using Tukey–Kramer HSD test.

### Training dataset minimum and maximum layer thickness

Qualitative assessment of observers’ thickness callipers showed no overlapping in repeated measurements, suggesting that measurements are not likely reproducible, as seen in Supplementary Fig. 3. Moreover, calliper over and undershoot was observed, which can lead to over and under measurement, respectively. In addition, the thickness vectors were not always perpendicular to the layer angle, hence prone to spurious results. Regardless of the lack of statistical differences in mean ONL and INL thickness between observers’ average and TT measurements, we analysed the minimum and maximum calliper values for each image.

**Figure 3:**
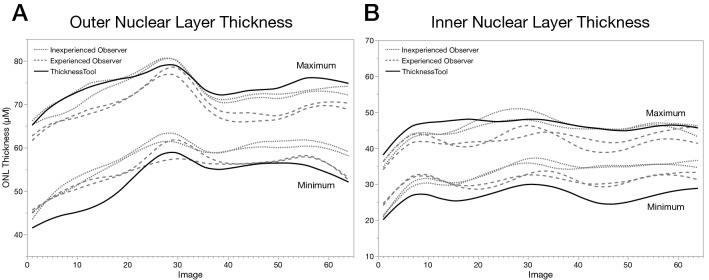
Parallelism plot of minimum and maximum retinal thickness measurements of observers and ThicknessTool. Results were fitted to a cubic spline with a default lambda of 0.05. (**A**) Outer nuclear layer. (**B**) Inner nuclear layer. Dotted lines represent the first and second measurements by an inexperienced observer. Dashed lines represent the first and second measurements by an experienced observer. Solid lines represent measurements by ThicknessTool (n = 64).

First, we constructed a parallelism plot of the minimum and maximum ONL and INL thickness calliper per image and observer. As seen in Fig. 3, the experienced observer had lower maximum and higher minimum ONL and INL values, without curve overlapping. Similarly, the inexperienced observer had higher minimum ONL and INL values without curve overlapping. These results suggest that experienced observers’ measurements are not reproducible. In addition, there were no significant differences between TT and any observers’ overall ONL minimum and maximum measurements (Table 2). However, we found significant differences across individual observer’s minimum and maximum measurements and between the inexperienced overall minimum INL and TT measurements (*p* < 0.001). In addition, the coefficient of variability between measurements was significantly different between inexperienced and observers’ average measurements for both ONL and INL (p < 0.001) and between the experienced observer and observers’ average measurements for the ONL (p < 0.009) (Supplementary Table 4). In summary, given the qualitative assessment of observers’ callipers’ lack of repeatability together with the quantitative analyses between manual callipers and a calibrated automated algorithm, results indicate that manual callipers measurements are not accurate nor reproducible.

**Table 2:** Training dataset minimum and maximum thickness measured by observers and ThicknessTool.

	Outer nuclear layer				Inner nuclear Layer			
	Minimum ± SD	*P* value*	Maximum ± SD	*P* value*	Minimum ± SD	*P* value*	Maximum ± SD	*P* value*
Inexperienced 1st	58.31 ± 8.76	**0.003**	73.94 ± 10.09	1.000	33.12 ± 7.30	** < 0.001**	45.44 ± 6.90	0.997
Inexperienced 2nd	57.35 ± 8.14	**0.031**	72.78 ± 10.06	0.987	33.39 ± 6.97	** < 0.001**	45.86 ± 6.91	1.000
Inexperienced overall	56.28 ± 8.54	0.218	75.00 ± 10.33	0.999	31.73 ± 7.12	** < 0.001**	46.96 ± 6.97	0.999
Experienced 1st	55.51 ± 8.19	0.534	70.03 ± 9.67	0.198	31.01 ± 5.88	**0.002**	42.45 ± 7.09	**0.048**
Experienced 2nd	54.60 ± 7.67	0.899	69.09 ± 8.53	**0.049**	30.95 ± 4.92	**0.002**	41.91 ± 7.35	**0.012**
Experienced overall	53.32 ± 7.83	0.999	71.34 ± 9.43	0.667	29.41 ± 4.89	0.207	44.53 ± 7.83	0.858
Observers overall	52.44 ± 8.07	1.000	75.74 ± 10.29	0.990	28.36 ± 5.52	0.812	47.92 ± 7.78	0.894
ThicknessTool	52.73 ± 8.20	–	74.30 ± 8.88	–	26.76 ± 5.23	–	46.28 ± 5.72	–

Values shown are means ± standard deviations.

*SD* standard deviation, *1st* first measurement, *2nd* second measurement.

*Comparisons for all pairs vs ThicknessTool using Tukey–Kramer HSD test.

### Agreement analysis between manual and thickness measurements

**Figure 4:**
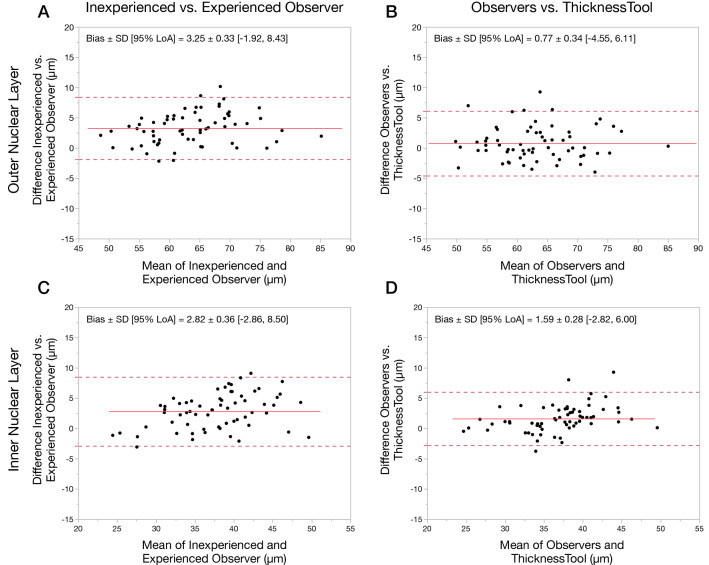
Agreement analysis of observers and ThicknessTool by Bland–Altman plots. Agreement between inexperienced and experienced average mean (**A**) ONL and (**C**) INL thickness. Agreement between observers and ThicknessTool (**B**) ONL and (**D**) INL thickness (n = 64). *SD* standard deviation, *LoA* limit of agreement.

We performed a bias analysis using Bland and Altman method^13^. For the ONL group, as seen in Table 3, we found statistically significant differences when comparing paired measurements, with a greater bias in inexperienced mean vs. experienced mean measurements (3.25 ± 0.33 µm, *p* < 0.001). In contrast, we found the lowest bias when comparing TT vs. observers’ means (0.77 ± 0.34 µm, *p* = 0.028). For the INL group, we found statistically significant differences when comparing inexperienced mean vs. experienced mean (*p* < 0.001), TT vs. inexperienced mean (*p* < 0.001), and TT vs. observers’ mean (*p* < 0.001). Most importantly, the bias between TT vs. observers’ INL mean (1.59 ± 0.28 µm) was lower than the those between any observers mean against each other (2.82 ± 0.36 µm). In addition, Bland–Altman plots displayed random variability within measurements (Fig. 4). In conclusion, these results indicate that ThicknessTool can measure ONL and INL with lower bias than observers’ average against each other for both ONL and INL.

**Table 3:** Training dataset agreement analysis of thickness measurements between observers and ThicknessTool.

	Bias ± SE	Lower 95% LoA	Upper 95% LoA	LoA interval	*P* value*
**Outer nuclear layer**					
Inexperienced 1st vs. inexperienced 2^nd^	0.86 ± 0.27	− 3.37	5.09	8.47	**0.002**
Experienced 1st vs. experienced 2nd	0.880 ± 0.36	− 4.76	6.52	11.29	**0.018**
Experienced mean vs. inexperienced mean	3.25 ± 0.33	− 1.92	8.42	10.35	** < 0.001**
ThicknessTool vs. inexperienced mean	2.40 ± 0.42	− 4.19	8.99	13.17	** < 0.001**
ThicknessTool vs. experienced mean	0.85 ± 0.34	− 4.48	6.18	10.66	**0.015**
ThicknessTool vs. observers mean	0.77 ± 0.34	− 4.56	6.10	10.66	**0.028**
**Inner nuclear layer**					
Inexperienced 1st vs. inexperienced 2nd	0.20 ± 0.27	− 4.03	4.43	8.47	0.465
Experienced 1st vs. experienced 2nd	0.38 ± 0.46	− 6.83	7.59	14.43	0.412
Experienced mean vs. inexperienced mean	2.82 ± 0.36	− 2.82	8.46	11.29	** < 0.001**
ThicknessTool vs. inexperienced mean	3.00 ± 0.40	− 3.27	9.27	12.54	** < 0.001**
ThicknessTool vs. experienced mean	0.18 ± 0.25	− 3.74	4.10	7.84	0.462
ThicknessTool vs. Observers mean	1.59 ± 0.28	− 2.80	5.98	8.78	** < 0.001**

*SE* standard error, *LoA* limit of agreement, *1st* first measurement, *2nd* second measurement.

*T-Test for matched pairs Values shown are means ± standard errors.

### Validation of thickness in a retinal detachment model

**Figure 5:**
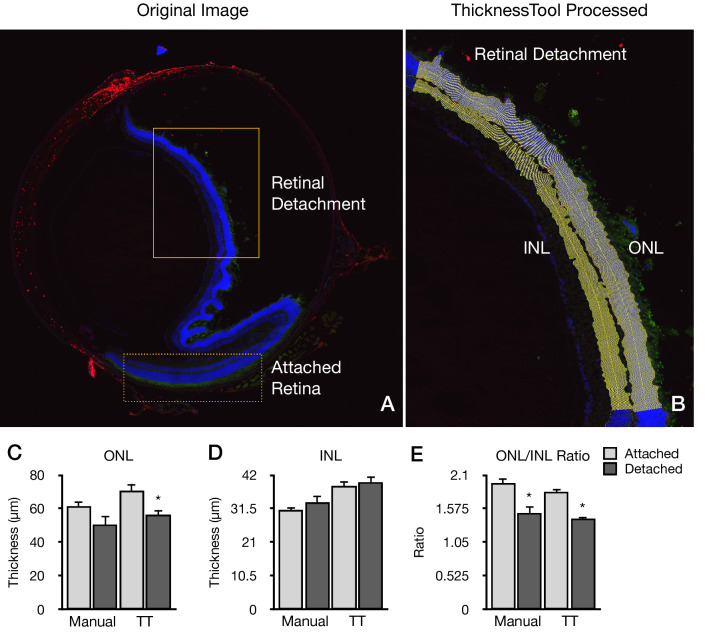
Validation of ThicknessTool in a retinal detachment model. (**A**) Representative image of mouse retina section of a retinal detachment displaying the attached (dotted line box) and detached retina (solid line box). (**B**) Representative image of ThicknessTool measurement of detached retina inner nuclear layer (INL) and outer nuclear layer (ONL) at 1-pixel interval callipers. (**C**) Outer nuclear layer (ONL) and (**D**) Inner nuclear layer (INL) thickness measurements from observer manual callipers and ThicknessTool in the attached and detached retina. (**E**) ONL/ONL + INL ratio measurements of observer manual callipers and ThicknessTool (n = 8 per group, *p ≤ .05).

To definitively validate the TT, we tested the tool in an experimental setting using a retinal detachment model, which causes photoreceptor cell death and subsequent ONL thinning and distortion (Fig. 5). We quantitated the ONL and INL thickness manually and using TT (Table 4). Interestingly, manual measurements failed to show significant ONL thinning in the detached retina (p = 0.069), in comparison to TT (p = 0.006). No differences were observed in the INL between the attached and detached retina by both methods as expected. Both methods showed significant differences in the ONL/INL ratio in the detached retina. These results suggest that TT can be more sensitive and detect significant true thinning beyond manual measurement capabilities. Collectively, these results suggest that TT is a sensitive, precise, and reliable tool to measure retinal nuclear layers in experimental models of disease.

**Table 4:** Validation dataset manual and ThicknessTool measurements in a retinal detachment model.

	Outer nuclear layer			Inner nuclear layer			ONL/ONL + INL Ratio		
	Attached	Detached	*P* value*	Attached	Detached	*P* value*	Attached	Detached	*P* value*
Manual	61.24 ± 8.39	50.47 ± 12.80	0.069	31.01 ± 2.58	33.43 ± 6.40	0.347	1.97 ± 0.22	1.51 ± 0.28	**0.003**
ThicknessTool	70.82 ± 10.04	56.14 ± 8.25	**0.006**	38.58 ± 4.34	39.79 ± 5.24	0.624	1.83 ± 0.16	1.41 ± 0.12	** < 0.001**

Values shown are means ± standard deviations.

*ONL* outer nuclear layer, *INL* inner nuclear layer.

*T-Test for independent samples.

### Application of thickness in digital images

**Figure 6:**
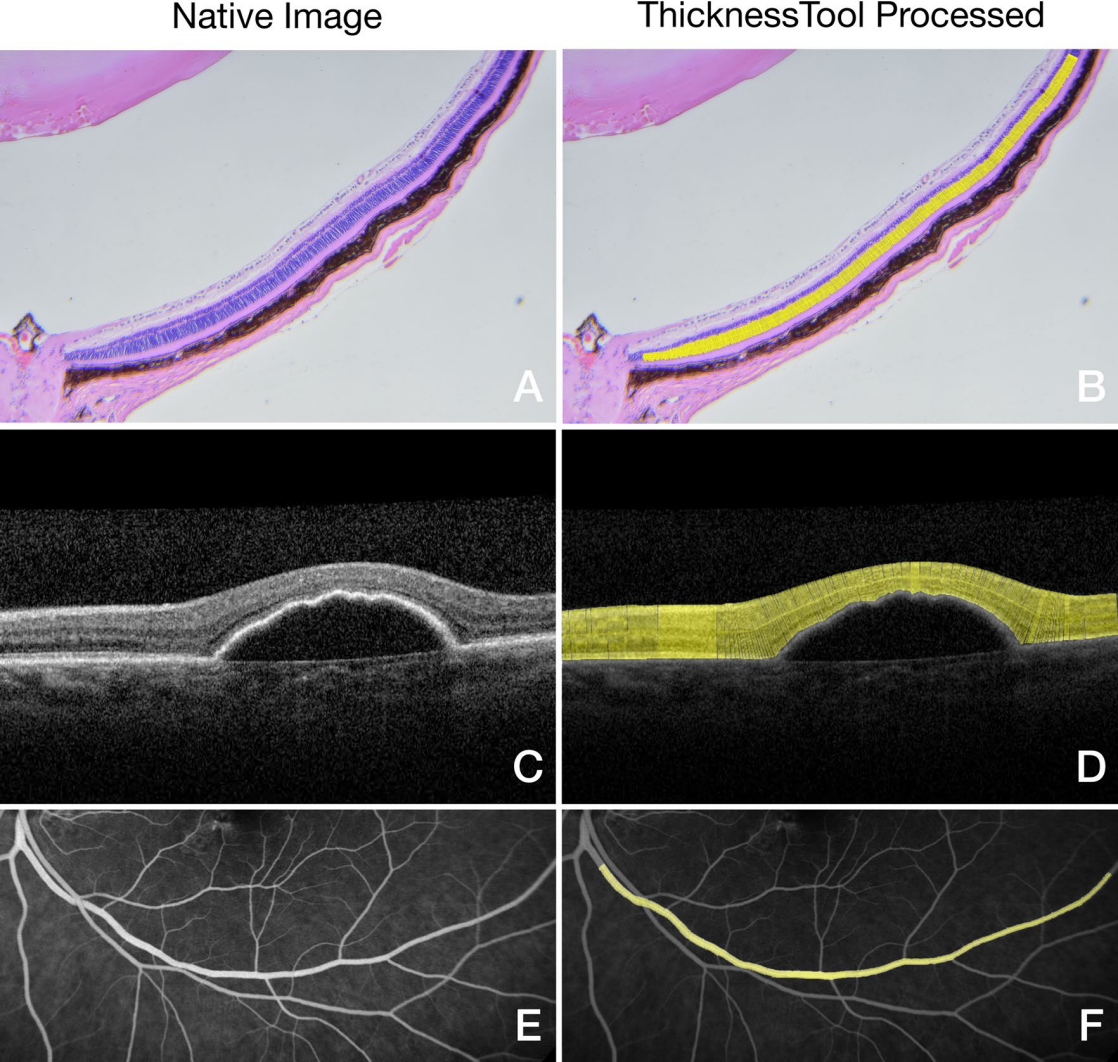
ThicknessTool application in digital images. (**A**,**B**) Haematoxylin–eosin staining of retinal cryosection. (**C**,**D**) Macular spectral-domain optical coherence tomography scan showing a pigment epithelial detachment in age-related macular degeneration. (**E**,**F**) Fluorescein angiogram of retinal vessels.

In order to examine the applicability of TT in different image modalities, we quantitated the thickness in various digital images. As seen in Fig. 6, TT was capable of quantitating the ONL thickness and corresponding profile in histology sections. Moreover, we challenged this tool in digital images of macular spectral-domain optical coherence tomography, with satisfactory results. Finally, we tested the performance in the quantitative assessment of retinal vessels in fluorescein angiogram images. The TT was able to draw accurate callipers with the correct vector in all cases. Collectively, these results indicate that ThicknessTool is a versatile tool with multi-platform capabilities.

## Discussion

In this study, we developed a sensitive, precise, and reliable tool to measure retinal layers. The performance of this tool was tested in calibration, training, and validation datasets with adequate and reliable performance. Collectively, results validate ThicknessTool for automated retinal thickness measurement. ﻿We speculate that this tool can aid in achieving precise, accurate, and fast measurements in multi-platform digital images.

In the development of new measurement tools, calibration images are mandatory to benchmark and finetune the performance of image processing algorithms to a standard^14^. We designed mock images with various known thickness ranging from 10 pixels (given a single nuclei size in our images) to 500 pixels (twice the outer nuclear layer measured in our dataset). In addition, we created a dataset with multiple mask rotations to mimic the random mask orientations. This step is necessary as TT callipers are constructed in a vectorial manner, in number and angles as the user or region of interest requires. Moreover, we tested TT capability to detect true thinning and thickening in a mock image with decreasing and increasing thickness, respectively. We found that the TT can automatedly and accurately measure thickness when challenged with different thicknesses and rotations.

Accurate assessment of photoreceptor cell death currently relies on manual counting by masked observers. Ideally, two masked two observers quantitate retina thickness, and their average is used to determine the overall outcome. We found that single observers’ measurements were prone to over and underestimation, regardless of their previous experience. In addition, observers draw incorrectly angled callipers, which can indirectly contribute to the abovementioned bias. Moreover, callipers were not located in corresponding locations in repeated same-image analysis, which dampens the reproducibility of the measurements. Altogether, this leads to a lack of accurate or reproducible retina thickness quantitation. This matter is of critical importance, as this bias can lead to α or β errors. For example, finding no difference in retinal thickness in cases where a neuroprotective treatment is effective, or even worse, finding a significant difference in retinal thickness in an ineffective treatment^15,16^. Such errors are not only misleading to the scientific community but also high priced.

Regardless of the performance of manual measurements to date, it remains the gold standard. For purposes of method agreement, we refrained from relying on mean comparison or correlation, as Bland and Altman have previously reported in their seminal work^12,17^. As authors describe, such agreement must be quantified, and cannot be assessed by mean comparison or hypothesis testing, as it does not exist in a present or absent binary form^13^. In addition, high correlation does not necessarily imply a high agreement between two methods, as it quantifies the degree to which two methods are related^18^. Agreement analysis showed lower bias of TT against observers than observers’ average against each other for both ONL and INL. Taken into consideration TT capability to detect true mask thinning and thickening, collectively, these results confer to TT a higher measurement agreement.

Moreover, the automated thickness analysis by TT yields a series of convenient metrics that remain hidden otherwise in manual measurements. On this note, Byun et al. have developed pioneering methods to estimate the ONL thickness exclusively^9^. However, their approach was not validated by thickness agreement analysis with manual observers. In their work, authors also described a method to quantify structural distortions in retinal tissue before and after injury, also known as distortion index. In essence, the authors stated that structural changes in the ONL are directly proportional to the distortion index. In our work, we estimated overall distortion in a similar approach using the index of dispersion (ID), calculated as the variance-to-mean ratio, as previously defined by Cox and Lewis^19^. Similarly, the index of dispersion allows the assessment of the overall calliper variation, which can provide detailed layer architecture data. Briefly, an ID of 0 represents no dispersion; therefore, we used the ID for calibration purposes, as mock images of known and constant thickness were uniform rectangles (ID = 0). We speculate that the index of dispersion can be equally used for further morphometric assessment.

Several limitations to this work should be considered. First, despite the calibration analysis performed beforehand, the validation of TT can only be performed against manual measurements. This process is cumbersome, as observers’ measurements carry an intrinsic bias. However, observers showed comparable performance, with overlapping confidence intervals. Second, TT analysis should be performed in areas distant from image edges, as skeleton are trimmed to avoid calliper-to-end image measurements. Despite the fact that TT allows skeleton and edge trimming, users should evaluate processed images for qualitative control. Third, TT measures thickness in binary segmented masks. Therefore, its performance is as strong as the segmentation algorithm used. In our case, we build this tool based on a previously validated algorithm for retina layer segmentation^4^. We recommend users to first tailor and validate their segmentation algorithm for optimal performance. Finally, regardless of the potential image modality application, this tool has been validated only for ONL and INL measurements in DAPI nuclei-stained murine retina cryosections. We believe future work can help expand the applicability of this tool for three-dimensional images, including upcoming imaging platforms.

In summary, we developed a fast, accurate, and precise retinal thickness measurement tool with multi-platform capabilities. In addition, the TT can be customized to user-defined preferences. This includes calliper number with a 1-pixel minimum distance between callipers, skeleton and edge trimming, and automated or manual layer segmentation. In addition, given the potential use with multi-platform capabilities, the source code can be easily modified to fit different applications. Finally, this tool is freely available to download and use. We expect this measurement tool could improve the outcomes and reduce bias in retina research.

## Supplementary information

Supplementary Information 1.

Supplementary Information 2.

Supplementary Information 3.

Supplementary Information 4.
