# Supplementary material for: ThicknessTool: automated ImageJ retinal layer thickness and profile in digital images
Source: Sci Rep. 2020 Oct 28;10:18459. doi: 10.1038/s41598-020-75501-y (PMC7595229; doi:10.1038/s41598-020-75501-y)
Supplement: Supplementary file 1 — Supplementary Information 1. [file 41598_2020_75501_MOESM1_ESM.docx]

ThicknessTool: Automated ImageJ Retinal Layer Thickness and Profile in Digital Images.

Daniel E. Maidana^1,2,3^, Shoji Notomi^1^, Takashi Ueta^1^, Tianna Zhou^1^, Danica Joseph^1^, Cassandra Kosmidou^1^, Josep Maria Caminal-Mitjana^3^, Joan W. Miller^1^, and Demetrios G. Vavvas^1,*^

^1^ From the Retina Service, Angiogenesis Lab, Massachusetts Eye and Ear Infirmary, Harvard Medical School, Boston, MA, USA
^2^ From the Department of Ophthalmology and Visual Sciences, University of Illinois at Chicago, Chicago, IL, USA
^3^ From the Retina Service, Bellvitge Hospital, University of Barcelona, Barcelona, Spain.

^*^ **Correspondence:** Demetrios G. Vavvas, MD, PhD
Massachusetts Eye and Ear Infirmary
243 Charles Street, Boston MA 02114, United States
Tel: +1 (617) 573-6874
e-mail: [Demetrios_Vavvas@MEEI.HARVARD.EDU](mailto:Demetrios_Vavvas@MEEI.HARVARD.EDU)

**Financial Disclosure:** The authors have no relevant financial relationships or interests to disclose.

**Funding Support:** This work was supported by Bayer Healthcare Global Ophthalmology Awards Program (DEM); the Yeatts Family Foundation (DGV, JWM); Loefflers family foundation (JWM, DGV); a Macula Society Research Grant award (DGV); a Physician Scientist Award (DGV) and an unrestricted grant (JWM) from the Research to Prevent Blindness Foundation; NEI Grant R21EY023079-01A1 (DGV); and NEI Grant EY014104 (MEEI Core Grant). The funders had no role in study design, data collection and analysis, decision to publish, or preparation of the manuscript.

**Running Head:** Automated Thickness Measurement in ImageJ

**Supplementary Tables:** 4 **Supplementary Figures:** 3 **Supplementary Code:** 1 **Supplementary Figure Legends**

Supplementary Table 1. Calibration dataset theoretical and ThicknessTool measurements.

Supplementary Table 2. Training dataset correlation analysis of outer nuclear layer thickness measurements.

Supplementary Table 3. Training dataset correlation analysis of inner nuclear layer thickness measurements.

Supplementary Table 4. Training dataset thickness measurement coefficient of variation between observers.

# Supplementary Figure Legends

**Supplementary Figure 1.** Capture of the graphical user interface dialog for the ThicknessTool.

**Supplementary Figure 2.** Outline of ThicknessTool (TT) Calibration. (A) Representative calibration image of a known thickness (200 pixels). (B) Representative calibration image after processing with thickness callipers (200 pixels). (C) Cropped area in a 45º tilted image showing even edges. (D) Cropped area in a 30º tilted image showing jagged edges. (E) Known vs. measured thickness in images of increasing mask size. (F) Known vs. measured thickness in a mock image with abrupt thinning and thickening.

**Supplementary Figure 3.** Qualitative assessment of manual and automated thickness measurements of outer (ONL) and inner nuclear (INL) retina layers. (A) Representative native image of mouse retinal section. (B) Inexperienced observer’s measurements, displaying a lack of reproducibility and oblique calliper vector (arrowheads). (C) Experienced observer’s measurements, displaying a lack of reproducibility and overshooting calliper vector (arrowhead). (D) ThicknessTool measurement of the INL and ONL at 1-pixel interval.

**Supplementary Code**

Supplementary Code 1. Source code of ThicknessTool.
