# Supplementary material for: ThicknessTool: automated ImageJ retinal layer thickness and profile in digital images
Source: Sci Rep. 2020 Oct 28;10:18459. doi: 10.1038/s41598-020-75501-y (PMC7595229; doi:10.1038/s41598-020-75501-y)
Supplement: Supplementary file 2 — Supplementary Information 2. [file 41598_2020_75501_MOESM2_ESM.pdf]

Supplementary Table 1. Calibration dataset theoretical and ThicknessTool measurements.

	**Theoretical**	**Overall (n = 21)**	**Straight/Even Edges (n = 5)**	**Jagged Edges (n = 16)**
Hypothesised Mean	-	200.00 ± 1	200.00 ± 1	200.00 ± 2
Mean Thickness	200.00 ± 0.00	199.88 ± 0.25	200.00 ± 0.00	199.85 ± 0.28
Minimum Thickness	200.00 ± 0.00	199.04 ± 0.74	200.00 ± 0.00	198.75 ± 0.74
Maximum Thickness	200.00 ± 0.00	200.57 ± 0.59	200.00 ± 0.00	200.75 ± 0.57
CoV Thickness	0.00 ± 0.00	0.00 ± 0.00	0.00 ± 0.00	0.00 ± 0.00
CoV Thickness (%)	0.00 ± 0.00	0.10 ± 0.09	0.00 ± 0.00	0.13 ± 0.07

CoV = coefficient of variation
Values shown are means ± standard deviations

Supplementary Table 2. Training dataset correlation analyses of outer nuclear layer thickness measurements.

	**Inexperienced 1st**	**Inexperienced 2nd**	**Inexperienced Mean**	**Experienced 1st**	**Experienced 2nd**	**Experienced Mean**	**Observers Mean**	**ThicknessTool**
Inexperienced 1st	-	0.96	0.99	0.90	0.91	0.92	0.97	0.88
Inexperienced 2nd	0.96	-	0.99	0.92	0.93	0.94	0.98	0.90
Inexperienced Mean	0.99	0.99	-	0.92	0.93	0.94	0.99	0.90
Experienced 1st	0.90	0.92	0.92	-	0.93	0.98	0.96	0.92
Experienced 2nd	0.91	0.93	0.93	0.93	-	0.98	0.97	0.90
Experienced Mean	0.92	0.94	0.94	0.98	0.98	-	0.98	0.93
Observers Mean	0.97	0.98	0.99	0.96	0.97	0.98	-	0.93
ThicknessTool	0.88	0.90	0.90	0.92	0.90	0.93	0.93	-

1st = first measurement; 2nd = second measurement
Values shown are Pearson correlation (*r*) coefficients (all coefficients $p < 0.001$)

Supplementary Table 3. Training dataset correlation analyses of inner nuclear layer thickness measurements.

	**Inexperienced 1st**	**Inexperienced 2nd**	**Inexperienced Mean**	**Experienced 1st**	**Experienced 2nd**	**Experienced Mean**	**Observers Mean**	**ThicknessTool**
Inexperienced 1st	-	0.94	0.98	0.86	0.80	0.88	0.97	0.86
Inexperienced 2nd	0.94	-	0.98	0.84	0.78	0.86	0.96	0.84
Inexperienced Mean	0.98	0.98	-	0.86	0.80	0.89	0.98	0.86
Experienced 1st	0.86	0.84	0.86	-	0.77	0.95	0.93	0.87
Experienced 2nd	0.80	0.78	0.80	0.77	-	0.94	0.89	0.87
Experienced Mean	0.88	0.86	0.89	0.95	0.94	-	0.97	0.92
Observers Mean	0.97	0.96	0.98	0.93	0.89	0.97	-	0.92
ThicknessTool	0.86	0.84	0.86	0.87	0.87	0.92	0.92	-

1st = first measurement; 2nd = second measurement
Values shown are Pearson correlation (r) coefficients (all coefficients $p < 0.001$)

Supplementary Table 4. Training dataset thickness measurement coefficient of variation between observers.

	**Outer Nuclear Layer**		**Inner Nuclear Layer**	
	Mean ± SD	*P* value*	Mean ± SD	*P* value*
Inexperienced	2.77 ± 2.52	**< 0.001**	4.82 ± 3.45	**< 0.001**
Experienced	3.81 ± 2.83	**0.009**	7.80 ± 6.42	0.602
Observers Average	5.39 ± 3.55	-	8.70 ± 5.55	-

SD = standard deviation; * = comparison for pair vs observers average using Tukey-Kramer HSD test
Values shown are means ± standard deviations
