# Supplementary material for: ThicknessTool: automated ImageJ retinal layer thickness and profile in digital images
Source: Sci Rep. 2020 Oct 28;10:18459. doi: 10.1038/s41598-020-75501-y (PMC7595229; doi:10.1038/s41598-020-75501-y)

Supplementary Figure 1

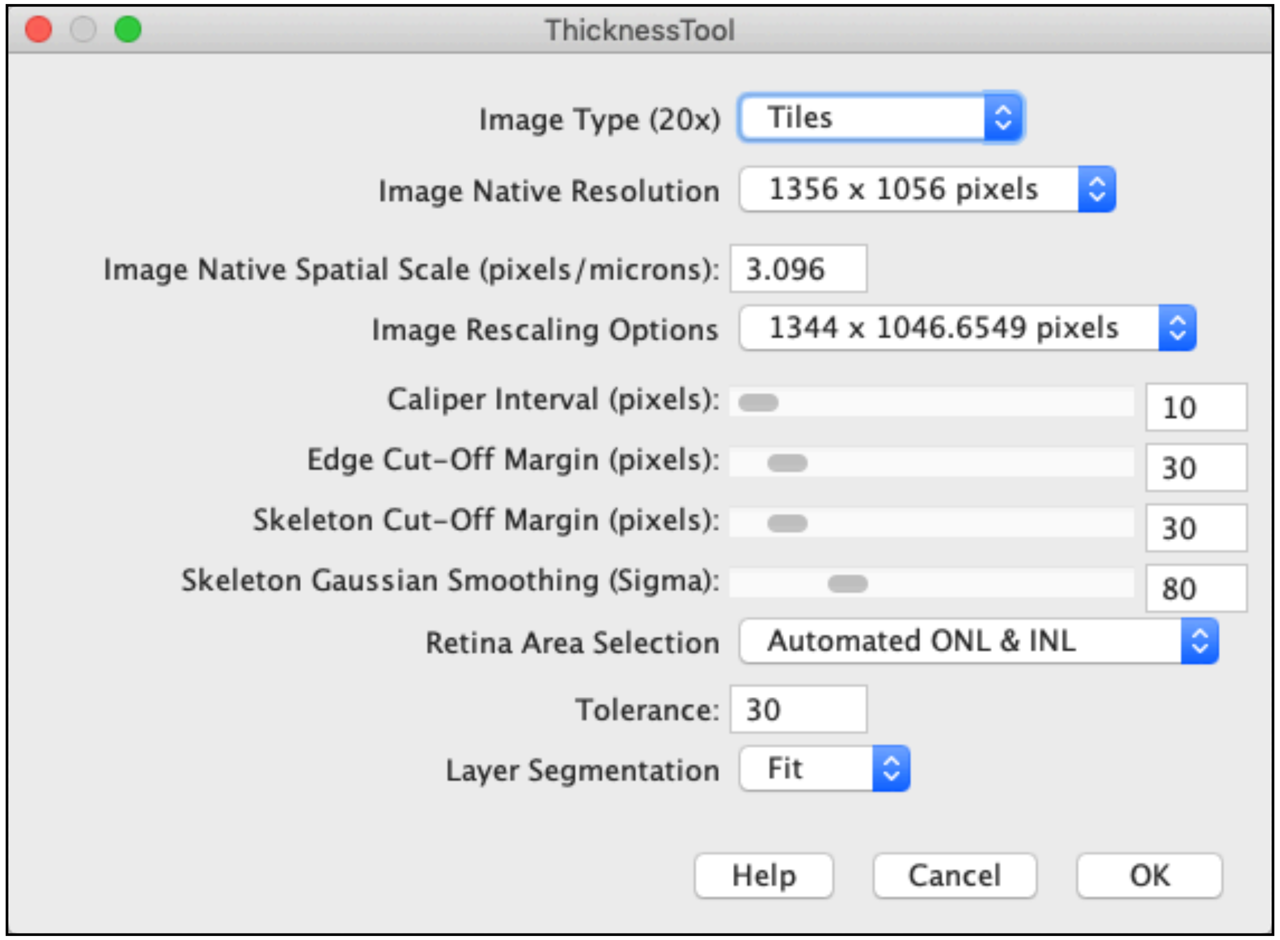

Supplementary Figure 2

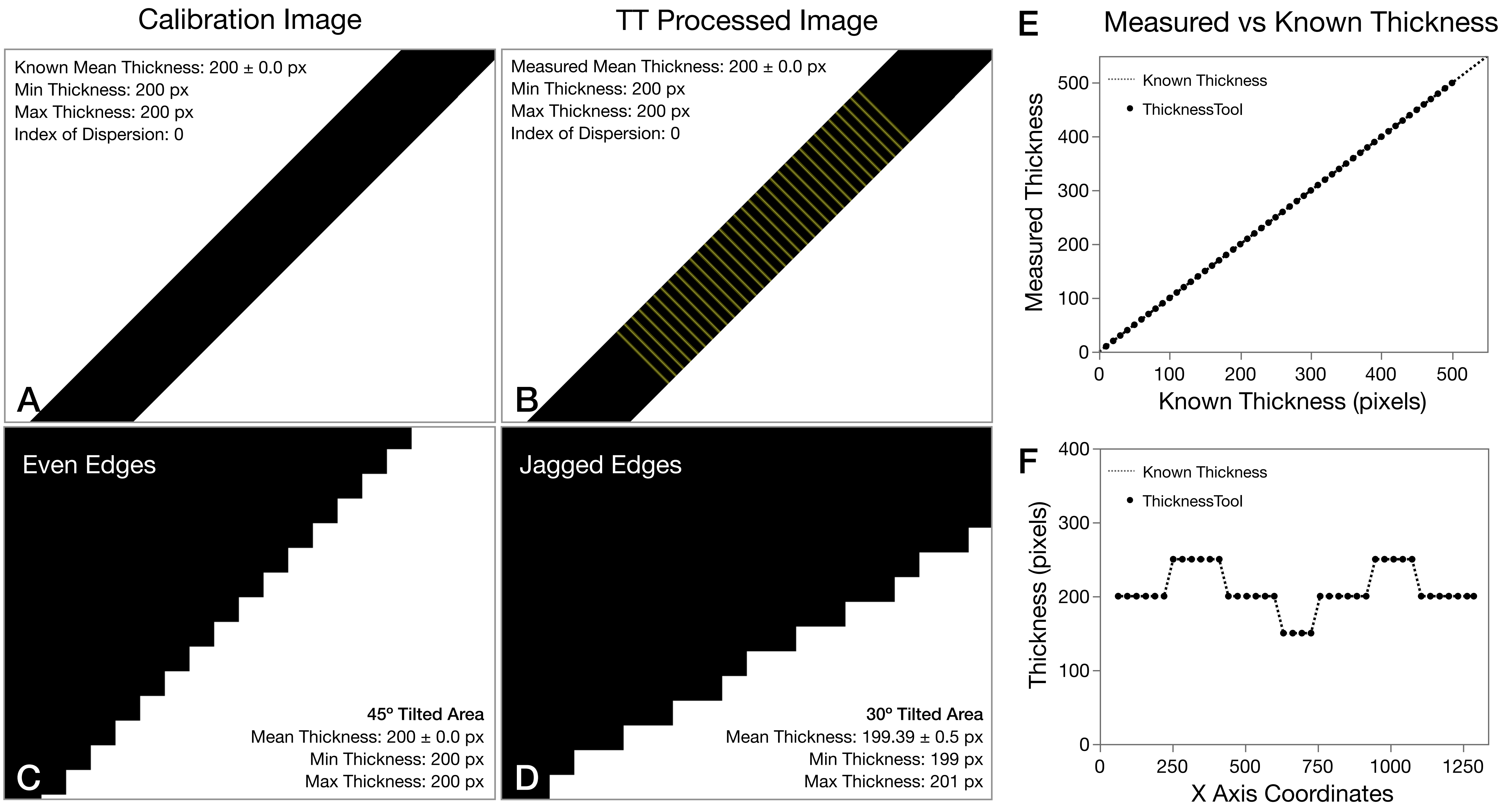

Supplementary Figure 3

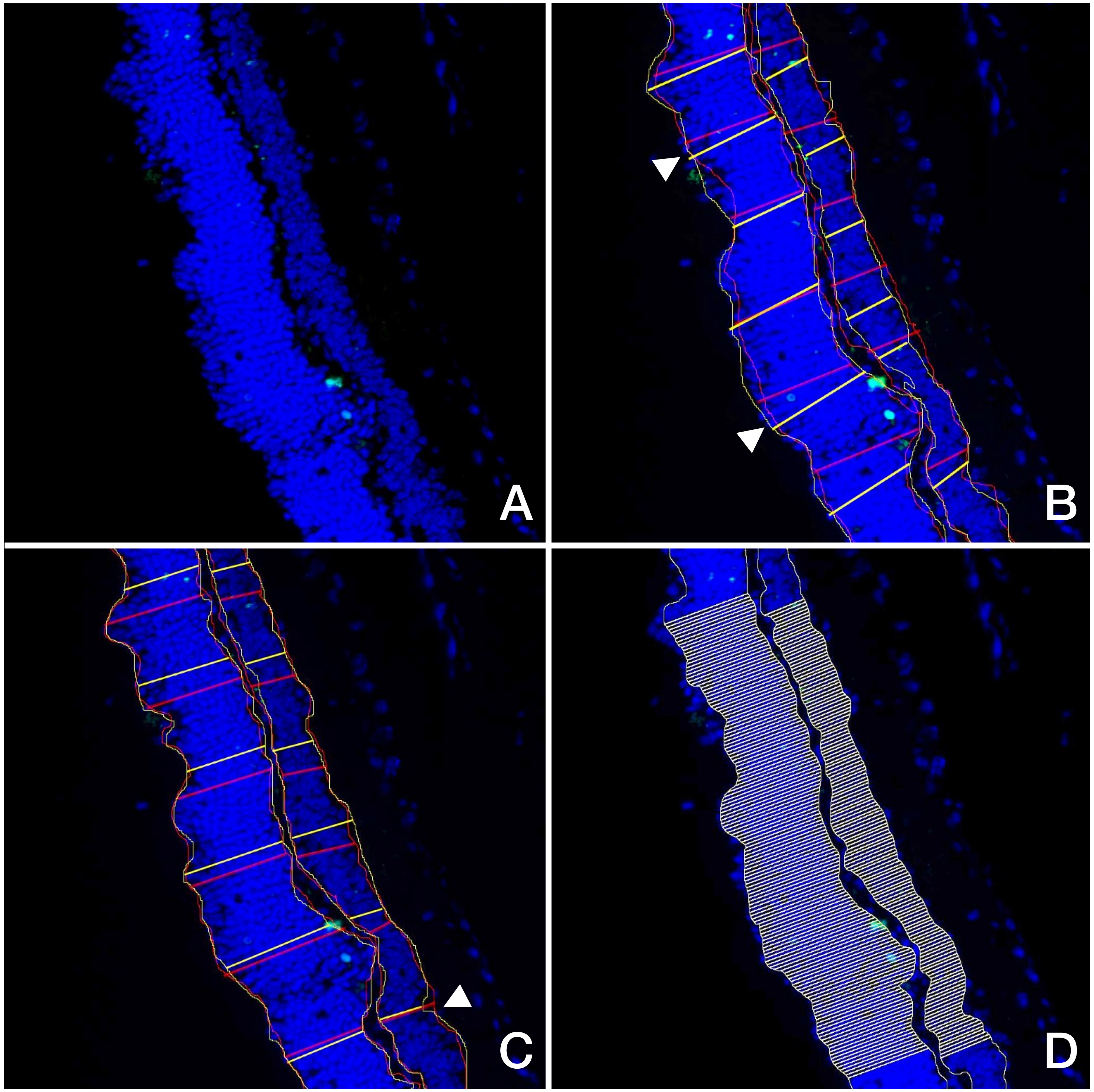

Supplement: Supplementary file 3 — Supplementary Information 3. [file 41598_2020_75501_MOESM3_ESM.pdf]
